# Bioavailability Study of Isothiocyanates and Other Bioactive Compounds of *Brassica oleracea* L. var. Italica Boiled or Steamed: Functional Food or Dietary Supplement?

**DOI:** 10.3390/antiox11020209

**Published:** 2022-01-22

**Authors:** Patrick Orlando, Ancuta Nartea, Sonia Silvestri, Fabio Marcheggiani, Ilenia Cirilli, Phiwayinkosi V. Dludla, Rosamaria Fiorini, Deborah Pacetti, Monica Rosa Loizzo, Paolo Lucci, Luca Tiano

**Affiliations:** 1 Department of Life and Environmental Sciences, Polytechnic University of Marche, 60131 Ancona, Italy; p.orlando@univpm.it (P.O.); s.silvestri@univpm.it (S.S.); f.marcheggiani@univpm.it (F.M.); r.fiorini@univpm.it (R.F.); l.tiano@staff.univpm.it (L.T.); 2 Department of Agricultural, Food and Environmental Sciences, Polytechnic University of Marche, 60131 Ancona, Italy; d.pacetti@univpm.it; 3 School of Pharmacy, University of Camerino, 62032 Camerino, MC, Italy; ilenia.cirilli@unicam.it; 4 Biomedical Research and Innovation Platform, South African Medical Research Council, Tygerberg 7505, South Africa; pdludla@mrc.ac.za; 5 Department of Pharmacy, Health and Nutritional Sciences, University of Calabria, 87036 Rende, CS, Italy; monica_rosa.loizzo@unical.it; 6 Department of Agri-Food, Animal and Environmental Sciences, University of Udine, 33100 Udine, Italy; paolo.lucci@uniud.it

**Keywords:** broccoli, carotenoids, glucosinolates, isothiocyanate, phylloquinone, steaming

## Abstract

The levels of bioactive compounds in broccoli and their bioavailability following broccoli intake can be affected by the cooking procedures used for vegetable preparation. In the present pilot study, we compared the human plasma bioavailability of antioxidant compounds (β-carotene, lutein and isothiocyanate) and of phylloquinone (vitamin K) on seven volunteers before and after the administration of boiled and steamed broccoli. Moreover, plasma isothiocyanate (ITCs) levels were also evaluated after the administration of a single dose of BroccoMax^®^, a dietary supplement containing GLSs with active myrosinase. Steam-cooking has been demonstrated to promote higher plasma bioavailability in ITCs than boiling (AUC_STEAMED_ = 417.4; AUC_BOILED_ = 175.3) and is comparable to that reached following the intake of BroccoMax^®^, a supplement containing glucoraphanin and active myrosinase (AUC = 450.1). However, the impact of boiling and steaming treatment on plasma bioavailability of lipophilic antioxidants (lutein and β-carotene) and of phylloquinone was comparable. The lutein and β-carotene plasma levels did not change after administration of steamed or boiled broccoli. Conversely, both treatments led to a similar increase of phylloquinone plasma levels. Considering the antioxidant action and the potential chemopreventive activity of ITCs, steaming treatments can be considered the most suitable cooking method to promote the health benefits of broccoli in the diet.

## 1. Introduction

*Brassica oleracea* represents one of the most important crops from the *Brassicaceae* family consumed in the human diet. Among the main cultivars, broccoli (var. italica) is one of the most used vegetables in Western cooking. They are reputed as “superfoods”, since they are rich sources of bioactive compounds whose health properties have been extensively studied [1]. In particular, they contain high levels of lipophilic antioxidant compounds, such as lutein, zeaxanthin and β-carotene, which are involved in cardiovascular diseases prevention and treatment [2,3,4]; vitamins (mainly K1 and C) [5]; hydrophilic antioxidant compounds (flavonoids, anthocyanins and phenolic acids) [6,7,8,9]; and glucosinolates (GLSs) [10]. Approximately 100 different GLSs are found in plants, and in broccoli, the most important are glucoraphanin, accounting for over 50% of the total; glucoiberin; glucoerucin; glucobrassicin; and neoglucobrassicin [11]. To become active, GLSs must be hydrolyzed to isothiocyanates (ITCs), a by-product of enzymatic synthesis by myrosinase, a β-thioglucosidase distributed in plant tissues but separately from the substrate. When tissues are disrupted due to food preparation or chewing, GLSs come in contact with the enzyme which breaks the β-thioglucose bond on the GLS. Myrosinase is also produced by gut microflora; therefore, hydrolysis can also be catalyzed following food ingestion [12,13]. Since the resulting aglycones are unstable, at neutral pH, ITCs formation is favored [14,15]. However, the presence of specifier proteins, such as epithio-specifier proteins (ESPs) and nitrile-specifier proteins (NSP), could promote a degradation of the unstable aglycones into epithionitriles and nitriles, respectively, reducing ITCs release [16].

After conversion from GLS, consumed ITCs are metabolized via the mercapturic acid pathway. In particular, by enzymatic activity of glutathione S-transferase (GST), ITCs react with GSH to produce GS conjugates. Subsequently, the conjugates undergo to sequential enzymatic modifications, first by γ-glutamyltranspeptidase (GT) to form cysteinylglycine-ITC conjugates, and then by cysteinylglycinase (CG) to form the cysteine-ITC conjugates, which are finally acetylated by *N*-acetyltransferase to produce *N*-acetyl-L-cysteine ITC conjugates; these are excreted in urine [17].

Since a relevant amount of experimental evidence has highlighted many beneficial effects of ITCs related to their antioxidant, antimicrobial, anti-inflammatory and anticancer activities [18,19,20,21], the total ITCs level in plasma is considered a good indicator of bioactive compounds responsible for beneficial effects related to Brassicaceae [22,23]. Among these, by far, the most thoroughly studied is sulforaphane, the ITC deriving from glucoraphanin hydrolysis, the most abundant GLS in broccoli. Due to its clinically confirmed biological efficacy [24,25,26,27,28], it is also an emerging compound in the nutritional supplements market, where its precursor is sometimes used in association with myrosinase or its isoform. 

In any case, the extension of the activities performed by bioactive molecules in the body is closely related to their bioavailability following consumption. In this context, preparation, storage and cooking methods could impact on the GLSs content in broccoli, as well as on potential ITCs released after the broccoli consumption. Generally, freezing is able to preserve most of the nutrients, thus ensuring a longer shelf-life preservation. Alanís-Garza et al. [29] found that freezing increased the extractability of total glucosinolates and the levels of carotenoids in broccoli with respect to raw counterpart. In accordance with this finding, González-Hidalgo et al. [30] stated that frozen broccoli retained glucosilonates. However, it is known that some thermal treatment could be deleterious for several nutrients, but, at the same time, it could enhance the extractability of others in relation to characteristics of the nutrients (chemical-physical, solubility, temperature-sensitivity, etc.). Thus, the choice of the cooking method should be function of the bioactive compound to be preserved or enhanced. In this sense, broccoli is rich in hydrophilic (i.e., GLS, polyphenols) and lipophilic (i.e., carotenoids) antioxidants, so it is very difficult to find a single suitable cooking method that minimizes the loss of all nutrients or promotes their availability. Generally, steam provides a more rapid and efficient heating than hot water (boiling). Steam-cooking was found to be a preferred procedure in order to preserve astaxanthin in fish meat [31] and to reduce the loss of GLS in broccoli and other brassica vegetables [32,33,34]. Nevertheless, the levels of lipophilic antioxidants (tocopherols and carotenoids) in cooked vegetables, including broccoli, resulted in being higher after boiling than steam-cooking [33,35,36]. However, it should be assessed if enhanced extractability of carotenoids by cooking leads to better bioavailability. The effect of different thermal treatments on the bioavailability of carotenoids from broccoli has been poorly investigated. Only a few authors have discussed plasma carotenoids after broccoli supplementation [2,37,38,39].

In this scenario, the present work represents a pilot study that aimed to evaluate how two methods commonly used for cooking broccoli (boiling and steaming) affect the bioavailability of vitamin K (phylloquinone) and of hydrophilic (ITCs) and lipophilic (β-carotene and lutein) antioxidants following a single acute consumption of vegetable. The levels of above bioactive compounds were assessed in raw and cooked broccoli and in the human plasma of seven healthy volunteers. Furthermore, the bioavailability of ITCs following a single acute consumption of broccoli was compared with those following an oral intake of a supplement containing glucoraphanin and active myrosinase.

## 2. Experimental Section

### 2.1. Chemicals and Reagents

Carotenoids standards (>95% purity; lutein and β-carotene), phylloquinone standard (≥99.0%), 1,2-benzendithiol (96%), 1,3-benzodithiol-2-thione standards (100%) and solvents HPLC grade (>95% purity, acetone, acetonitrile, dichloromethane, ethanol, methanol and water) were purchased by Merck (Darmstadt, Germany). Potassium phosphate and acetate ammonium were purchased by ITW Company (Darmstadt, Germany). Milli-Q water was purified with a Millipure System (Milford, CT, USA).

### 2.2. Broccoli Sampling and Cooking Treatments

Fresh broccoli (*Brassica oleracea* L. var. italica) was purchased from a local farm (Agrinovana, Petritoli, Italy). Within 24 h of collection, the vegetables were selected, cleaned and deprived of the stalks and stems. Since the content of GLSs and carotenoids in broccoli is subject to variation, due to environmental conditions, only broccoli derived from a single crop was included in the study, and the florets had to weigh 10.0 ± 2.0 g and be 2.5 ± 0.5 cm long. Portions of 200 g of broccoli were prepared and frozen at −20 °C for 15 days before cooking. Two different thermal treatments were applied to the broccoli: boiling and steaming. The first was achieved by using a pot measuring 18 cm in diameter with a lid, containing 1.5 L of unsalted water and heated on an induction hob. Then 200 g of selected broccoli florets was added to boiling water and cooked for 10 min. Steaming was performed in an oven (Steam oven, AEG, Electrolux) by steam injection in the chamber (RH% = 100), and the same amount of broccoli was cooked at 99 °C for 13 min, after pre-heating of the oven. Both cooking-treatment conditions were chosen in line with real household conditions. Preliminary tests were performed to select the most suitable conditions for ensuring a satisfactory level of cooking of broccoli in order to be eaten by the volunteers.

### 2.3. Study Design 

A pilot study was designed. Seven healthy subjects (3 male/4 female) volunteered for the three-way crossover. A pilot study was designed. Due to the limited number of subjects, in order to minimize the age-related effects on the experimentation, a narrow age group of the subject (25–35 years) was considered. The inclusion criteria were not taking vitamin and mineral supplements or fortified food in the last month, no smoking, BMI within 18.5–25 kg/m^2^ and being aged between 25 and 35 years. The exclusion criteria were diagnosed diseases, such as allergies, diabetes, gastrointestinal and renal disease; vegetarian diet; and none of the female subjects was pregnant or lactating. Moreover, participants were recommended to limit the consumption of the following food rich in carotenoids, vitamin K and GLSs at least a week before the trial: broccoli, cauliflower, kale, Brussels sprouts, cabbage, rocket, horseradish, turnip and mustard; corn, potatoes, carrots, peppers, yolk egg, pumpkin, spinach, chard, chicory, red radish, romaine lettuce, tomatoes, asparagus, avocado, peas, soybean, oranges, apricots, pistachio and kiwi fruit.

The study was conducted according to the guidelines of the Declaration of Helsinki and approved by Departmental Review Board of Polytechnic University of Marche (protocol code 2020-0614 B43). Informed consent was obtained from all subjects involved in the study.

### 2.4. Dietary Intervention and Biological Sample Collection

In the morning, at fasting, a single dose of 400 g of boiled broccoli or steamed broccoli, or a single dose including 3 capsules of BroccoMax^®^ (Jarrow Formulas, California, LA, USA) containing glucoraphanine and activated myrosinase was consumed by each of 7 subjects on three separate occasions in a randomized manner, alternating with at least 2 weeks of washout period.

Blood samples (6 mL) were withdrawn from fasting volunteers and collected in ethylenediaminetetraacetic acid (EDTA) and heparin vacutainers before and after 3, 6, 12 and 24 h from supplementation. Collected blood was centrifuged at 1500× *g* for 15 min at 4 °C to separate plasma and kept at −80 °C until analyses.

### 2.5. Total GLS in Broccoli as ITCs Equivalents and ITCs Plasma Level

In this study, total GLSs in broccoli is expressed as ITCs equivalents released by a thioglucosidase, as suggested by Conaway et al. [22]. In fact, after extraction of GLSs from florets, obtained by homogenizing 50 mg of minced broccoli with 0.2 mL of deionized water, followed by 3 cycles of vortex (30 s) and sonication (3 min), the GLSs were hydrolyzed to ITCs by incubating for 2 h in the dark at 60 °C with 2 mg *Sinapis alba thioglucosidase* (Sigma-Aldrich, St. Louis, MO, USA), as described by Atwell et al. [40]. Moreover, total ITCs equivalent in broccoli and the ITCs plasma level were quantified by using a cyclocondensation reaction developed by Ye et al. [41], according to which ITCs react with the sulfhydryl groups of 1,2-benzendithiol, producing 1,3-benzodithiol-2-thione, which is quantified chromatographically at 365 nm. Briefly, broccoli extract or plasma (0.2 mL) was incubated at 65 °C for 2 h with 0.2 mL of 100 mmol/L potassium phosphate buffer, pH 8.5 and 0.4 mL of 20 mmol/L 1,2-benzenedithiol in acetonitrile [41]. After cooling to room temperature and centrifugation at 1200× *g* for 5 min, 40 µL of supernatant was injected into the reverse phase HPLC column (Kinetex, C18 100 A, 250 mm × 4.6 mm i.d., 5 µm, Phenomenex, Torrance, CA, USA) and eluted with 80% methanol/20% water (by volume) at a flow rate of 2 mL/min. The 1,3-benzodithiole-2-thione was eluted at about 5 min, and the peak was quantified by using an HPLC system (YL Instrument, Anyang, Korea) that was equipped with UV–visible detector (YL Instrument 9300) set at 365 nm [41], a column compartment at 35 °C (YL Instrument 9330) and an autosampler (YL Instrument 9150) and driven by YL-Clarity software™. The instrument was calibrated with pure 1,3-benzodithiol-2-thione standard, and a good correlation coefficient (R^2^) of 0.996 was obtained in the range of 22–1413 ng/mL.

### 2.6. Carotenoids in Broccoli and Plasma

Carotenoids in broccoli were extracted in accordance with Nartea et al. [36]. A total of 100 mg of freeze-dried broccoli powder was extracted twice by adding acetone (5 mL, 4 °C), vortexing and keeping at 4 ± 1 °C (15 min), shaking (5 min) and centrifuging (1370 rpm, 10 min, 4 °C). The supernatant was filtered (Sartorius regenerate cellulose 0.45 μm) and taken to dryness at 30 °C with rotavapor and resuspended in 0.5 mL of acetone for injection (2 μL).

Carotenoids in plasma were extracted as reported by Chauveau-Duriot et al. [42]. Plasma (350 μL) was deproteinized with the same volume of ethanol, and carotenoids were extracted twice with *n*-hexane (800 μL). The mixture was vortexed (30 s) and centrifuged (1370× *g* for 10 min at 4 °C). Organic phases were collected and evaporated under nitrogen, and the residue was dissolved in acetone (100 μL) for injection (2 μL).

Carotenoids analysis was run on and Acquity Ultra Pressure Liquid Chromatographic H-class System (Waters Corporation, Milford, CT, USA) that was equipped with a Photodiode Array Detector (PDA) and driven by Empower software v2.0, as reported by Nartea et al. [36]. A faster version (20 min instead of 46 min) of the method developed by Chauveau-Duriot et al. [42] was applied by using an Acquity column UPLC BEH C18 (2.1 mm × 100 mm, 1.7 μm). The mobile phase was composed of phase A, consisting of acetonitrile (75%), dichloromethane (10%) and methanol (15%); and phase B, consisting of ammonium acetate in water (0.05 M). Gradient was started at 75:25 (A:B) to 10 min, 98:2 (A:B) from 10 to 11 min and 98:2 (A:B) till 20 min. Flow rate was 0.4 mL/min, column oven was set at 35 °C and sample loading was carried out at 20 °C. PDA analysis was performed at 450 nm wavelength on a spectrum scanning in the 210–500 nm range. Carotenoids were identified by comparison of retention time and absorbance spectrum with pure standards and quantified by external calibration. Good correlation coefficients (R^2^) of 0.999 were obtained in the range of 1–100 µg/mL for lutein and 0.05–100 µg/mL for β-carotene.

### 2.7. Phylloquinone in Broccoli and Plasma

Phylloquinone was extracted from broccoli in two ways: by homogenizing 50 mg of minced florets with 1 mL of ethanol, followed by 3 cycles of vortex (30 s) and sonication (3 min); and from plasma, by adding 250 µL of ethanol to 50 µL of sample and vortexing vigorously. After centrifugation (1200× *g* for 5 min and 12,900× *g* for 2 min, respectively), 40 µL of surnatant was injected into the column (Kinetex, C18 100 A, 100 mm × 4.6 mm i.d., 2.5 µm, Phenomenex, Torrance, CA, USA) connected to a post-chromatographic reducing column (CQ-R 2.0 × 20 mm, Shiseido) by using an HPLC system (9300, YL Instrument, Anyang, Republic of Korea) equipped with a fluorescence detector (Nanospace SI-2, Shiseido), as described by Cirilli et al. [43]. The optimized detection wavelengths were 335 nm (excitation) and 430 nm (emission). Phylloquinone peak showed a retention time of 8.5 min, and its level in plasma and broccoli was quantified by using a pure external standard of phylloquinone, showing a good correlation (R^2^ > 0.999) in a range between 0.1 and 6.25 µg/mL.

### 2.8. Statistical Analysis

Each plasma and broccoli sample was analyzed in three technical and five biological replicates respectively, and the results are expressed as mean ± standard deviation (SD). Plasma bioavailability was expressed as Area Under Curve (AUC) and, for each time point, as mean ± SD. The significance of differences among the samples were evaluated by using one-way ANOVA with Tukey’s multiple comparison test if significant. Statistical analysis was performed by using GraphPad Prism^®^ 6.0 Software.

## 3. Results

### 3.1. ITCs Equivalent, Phylloquinone, Lutein and β-carotene in Broccoli 

#### 3.1.1. ITCs Equivalent

As above reported, the total GLSs levels in broccoli are expressed as ITCs equivalent and quantified chromatographically at 365 nm as 1,3-benzodithiol-2-thione after cyclocondensation reaction.

**Figure 1:**
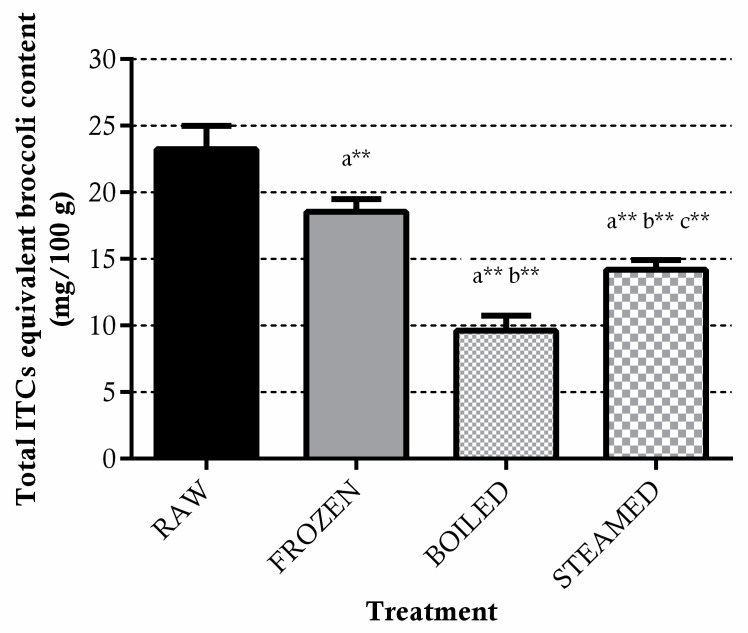
Total ITCs equivalent levels in raw, frozen (−20 °C for 15 days) boiled and steamed broccoli. All samples were incubated with myrosinase at 60 °C for 2 h to promote the hydrolysis of GLSs to ITCs. Data are expressed as mg/100 g of fresh vegetable ± SD of five cooking replicates. ** *p* < 0.01 vs. raw (a), frozen (b) and boiled (c).

The total level of ITCs equivalent quantified in raw broccoli was 23.3 ± 1.6 mg/100 g (Figure 1). Freezing and both cooking procedures significantly decreased the total ITCs equivalent content in broccoli (ITCs eq_frozen_ = 18.5 ± 0.9 mg/100 g, ITCs eq_steamed_ = 14.2 ± 0.7 mg/100 g, ITCs eq_boiled_ = 9.6 ± 1.1mg/100 g; *p* < 0.01); however, the steaming treatment was able to better preserve these bioactive molecules by limiting their loss as compared to boiling (*p* < 0.01).

#### 3.1.2. Phylloquinone

**Figure 2:**
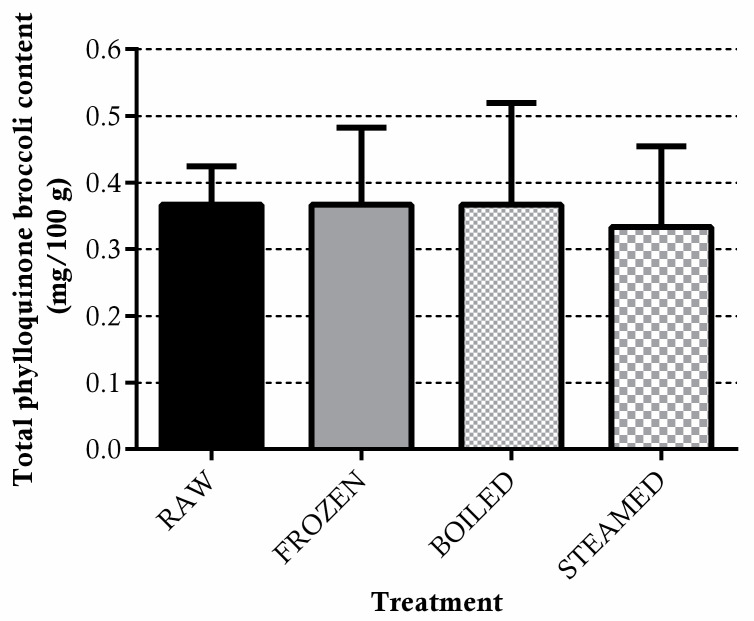
Total phylloquinone levels in raw, frozen (−20 °C for 15 days) boiled and steamed broccoli. Data are expressed as mg/100 g of fresh vegetables ± SD of five cooking replicates.

Conversely, freezing and cooking treatments did not affect the phylloquinone content in broccoli. In fact, as shown in Figure 2, the amount of phylloquinone extracted from raw, frozen and cooked vegetables was analogous (K1_raw_ = 0.35 ± 0.01 mg/100 g, K1_frozen_ = 0.35 ± 0.09 mg/100 g, K1_steamed_ = 0.35 ± 0.18 mg/100 g and K1_boiled_ = 0.34 ± 0.06 mg/100 g).

#### 3.1.3. Carotenoids

**Figure 3:**
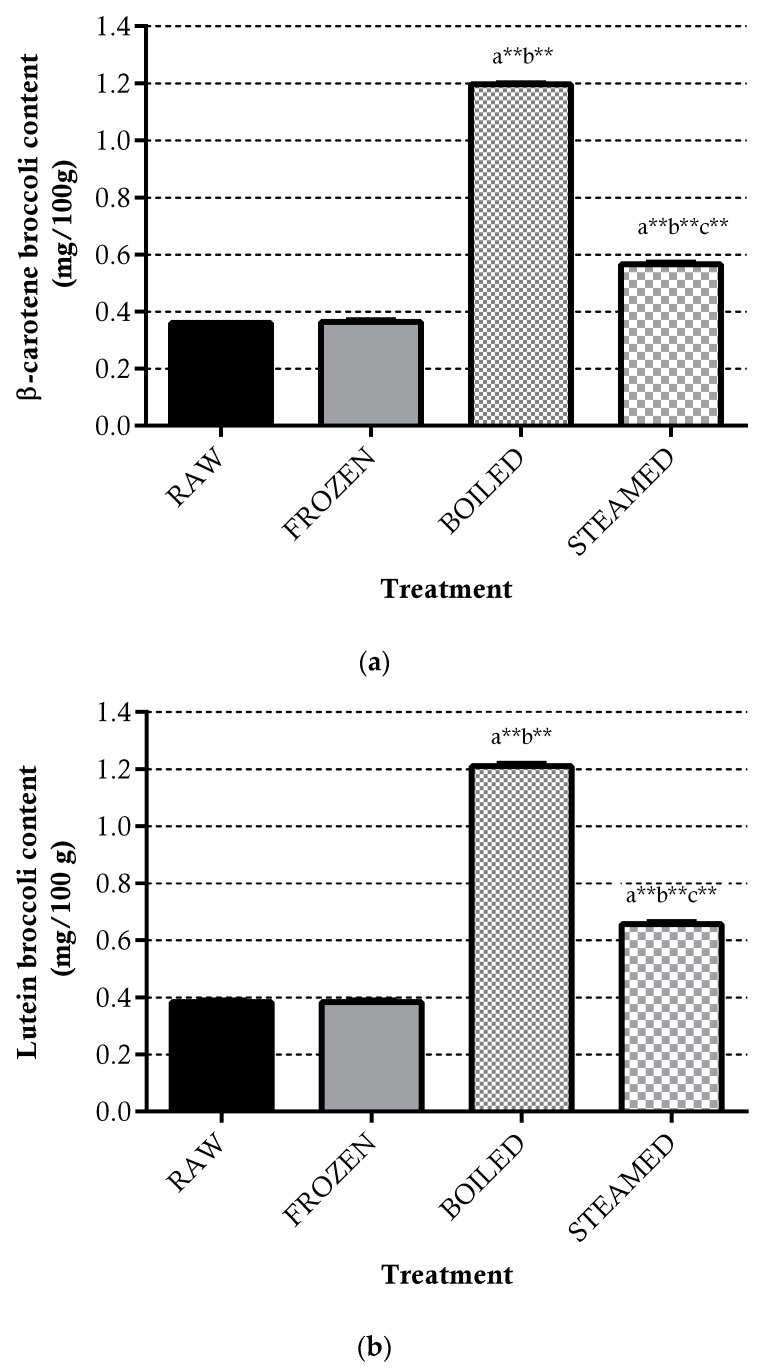
Total β-carotene (**a**) and lutein (**b**) levels in raw, frozen (−20 °C for 15 days) boiled and steamed broccoli. Data are expressed as mg/100 g of fresh vegetables ± SD of five cooking replicates. ** *p* < 0.01 vs. raw (a), frozen (b) and boiled (c).

The carotenoids profiles in raw and cooked broccoli are reported in Figure 3a,b. In raw broccoli, lutein (non-provitamin A carotenoid) and β-carotene, displaying provitamin A activity, were found in the same amount (0.37 mg/100 g broccoli). Freezing did not affect the content of both carotenoids in broccoli, while both thermal treatments increased the extractability of lutein and β-carotene. 

Boiling resulted in being more efficient as a treatment for carotenoids extraction as compared to steaming, as β-carotene was found to be 3.3-fold (boiling) and 0.9-fold (steaming) more in cooked than in the raw-vegetable states (*p* < 0.01) and lutein was found to be 2.4-fold (boiling) and 1.3-fold (steaming) more enhanced after cooking.

### 3.2. Plasma Bioavailability of Bioactive Compounds 

The plasma bioavailability of ITCs, phylloquinone, β-carotene and lutein was assessed after an acute intake of 400 g of boiled or steamed broccoli, and the intake of these bioactive compounds is summarized in Table 1. The ITC plasma bioavailability was evaluated by comparing cooked vegetables with the supplement BroccoMax^®^, containing glucoraphanin and active myrosinase.

**Figure 4:**
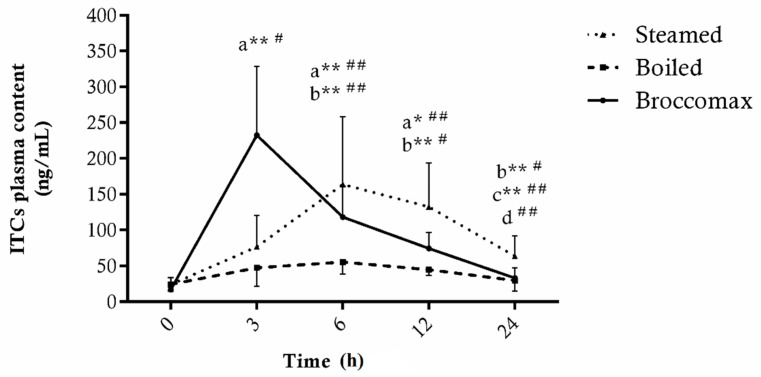
Total isothiocyanates (ITCs) levels in plasma of 7 subjects before (0) and after 3, 6, 12 and 24 h consumption of supplement (BroccoMax^®^) and broccoli cooked with boiling or steaming treatment. Data are expressed as ng/mL of plasma ± SD of three technical replicates. * *p* < 0.05 and ** *p* < 0.01 vs. 0 h (a), 3 h (b) and 6 h (c) in subjects supplemented with BroccoMax^®^; ^#^ *p* < 0.05 and ^##^ *p* < 0.01 vs. 0 h (a), 3 h (b), 6 h (c) and 12 h (d) in subjects supplemented with steamed broccoli.

In Figure 4, the plasma levels of ITCs at the baseline and after 3/6/12/24 h of supplementation with three capsules of BroccoMax^®^ or 400 g of boiled or steamed broccoli are reported. The supplement intake promoted the highest plasma ITC levels in terms of both the AUC (BroccoMax^®^ = 450.1) and C_max_ achieved 3 h later (BroccoMax^®^_3h_ = 232.4 ± 96.2 ng/mL). From 6 h after supplementation, the ITCs plasma levels significantly decreased (BroccoMax^®^_6h_ = 118.1 ± 43.4 μg/mL, *p* < 0.01), reaching levels statistically not different compared to baseline after 24 h (BroccoMax^®^_baseline_ = 17.3 ± 3.3, BroccoMax^®^_24h_ = 33.2 ± 14.5 μg/mL, *p* = 0.92).

However, steamed broccoli resulted in a slightly lower but statistically not significant AUC compared to the supplement (Steamed = 417.1, *p* = 0.98) and, differently from BroccoMax^®^, the maximum levels of ITC were reached 6 h after ingestion and remained high up to 12 h (Steamed_6h_ = 164.0 ± 94.9 ng/mL, Steamed_12h_ = 132.6 ± 60.7 ng/mL). 

Conversely, the consumption of boiled broccoli did not significantly affect the plasma level of these molecules over the next 24 h, reaching an AUC of 175.3, which is significantly lower than that obtained with steamed (*p* = 0.017) and BroccoMax^®^ (*p* = 0.016).

**Figure 5:**
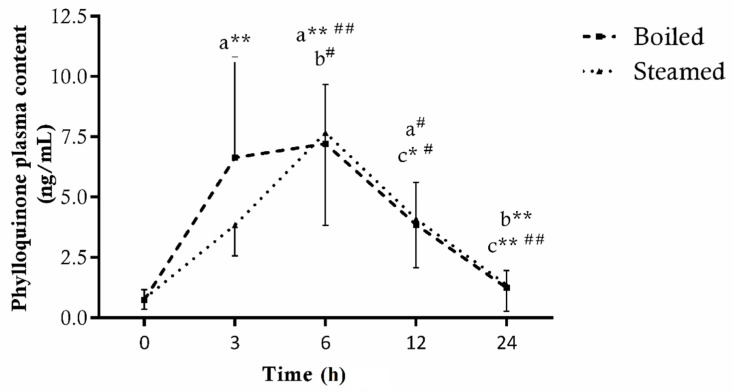
Total phylloquinone levels in plasma of 7 subjects before (0) and after 3, 6, 12 and 24 h consumption of broccoli cooked with boiling or steaming treatment. Data are expressed as ng/mL of plasma ± SD of three technical replicates. * *p* < 0.05 and ** *p* < 0.01 vs. 0 h (a), 3 h (b) and 6 h (c) in subjects supplemented with boiled broccoli; ^#^ *p* < 0.05 and ^##^ *p* < 0.01 vs. 0 h (a), 3 h (b) and 6 h (c) in subjects supplemented with steamed broccoli.

Moreover, eating 400 g of broccoli increased the plasma levels of phylloquinone independently from the heat treatment used. In fact, as reported in Figure 5, the trend of both curves is very similar, except that the consumption of boiled broccoli led to a significant rise in vitamin K1 already 3 h later (Boiled_baseline_ = 0.8 ± 0.4, Boiled_3h_ = 6.6 ± 4.2 ng/mL, *p* < 0.01). However, there were no differences in the pharmacokinetic parameters between the two cooking methods, as the plasma bioavailability achieved following boiled and steamed broccoli administration showed AUC values of 18.7 and 16.7 (*p* = 0.84), with a C_max_ at 6 h of 7.2 ± 2.5 ng/mL and 7.7 ± 3.8 ng/mL, respectively. 

**Figure 6:**
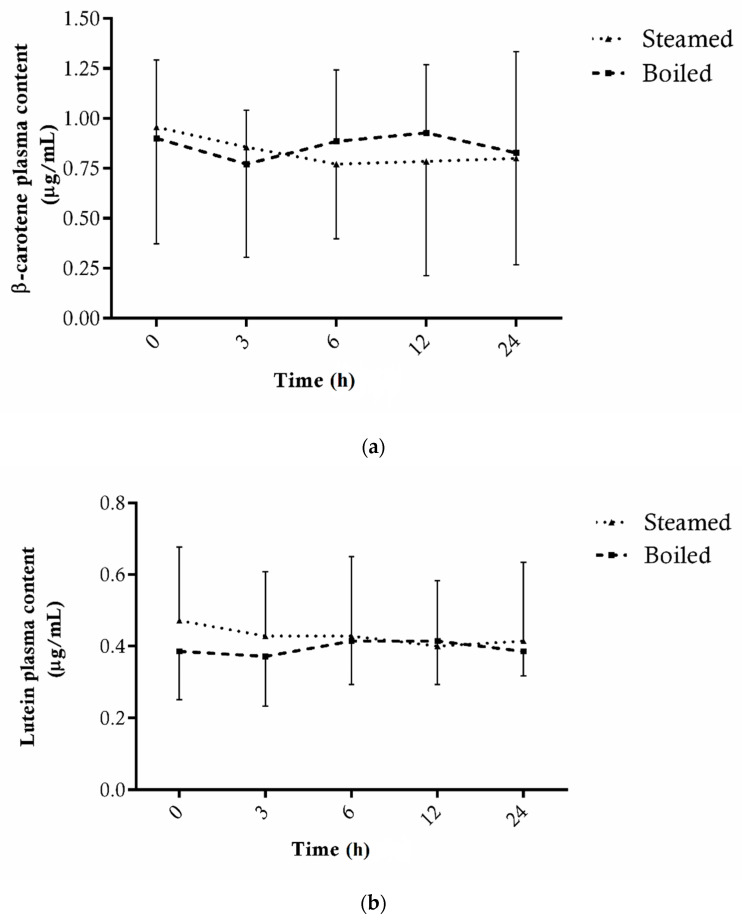
Total β-carotene (**a**) and lutein (**b**) levels in plasma of 7 subjects before (0) and after 3, 6, 12 and 24 h consumption of broccoli cooked with boiling or steaming treatment. Data are expressed as µg/mL of plasma ± SD of three technical replicates.

Conversely, the plasma carotenoid levels analyzed in this study were not affected by broccoli. In fact, as shown in Figure 6a,b, the amount of both lutein and β-carotene quantified showed non-significant fluctuations during the 24 h following supplementation.

## 4. Discussion

Bioactive compounds are essential and non-essential molecules found in fruit and vegetables that are able to modulate metabolic processes, resulting in the improvement of human health [44]. In the last decades, several studies have focused on the beneficial role of ITCs, the active compounds deriving from the GLSs mainly present in Brassicaceae by hydrolytic activity of myrosinase. However, the different methods of processing and cooking of foods can alter the amount and the availability of these compounds, thus affecting their bioavailability and, consequently, their biological role.

According to our results, both boiling and steaming treatments significantly decreased total ITCs equivalent level in florets of broccoli; steaming minimized their loss (−23% compared to frozen florets), in contrast to boiling, which almost halved them (−48%). GLSs are water-soluble compounds, and, therefore, they are usually lost during conventional cooking, because of leaching into surrounding water, due to cell lysis [32]. The ability of steaming to minimize GLS losses compared to boiling and other thermal treatments has been confirmed in several publications, albeit with variable data [45,46,47]. In fact, Baenas et al. [47] found GLS loss of more than 85% and 50% in boiled and steamed broccoli, respectively. However, in a study of Vallejo et al. [45], steaming had a minimal effect on these molecules, while boiling had a loss rate of 55%. According to previous reports, the vegetable matrix, myrosinase activity, the presence of specific proteins and extrinsic postharvest represent determining factors on the degradation of compounds during food processing [48]. Yuan et al. [46] pointed out that the variation in GLSs levels following different cooking methods of broccoli also depends on the composition of the aglycon side chain, after demonstrating that the total aliphatic glucosinolates remained almost unchanged in steamed broccoli and decreased by 41% in boiled, while the content of total indole glucosinolates decreased in both steamed (37%) and boiled (60%) broccoli.

The present study highlighted the crucial role of cooking procedures on plasma bioavailability of ITCs upon broccoli uptake. In fact, the intake of 400 g of steamed broccoli promoted a much higher ITC plasma adsorption than boiled vegetables. This is partly due to both GLSs (precursors of ITC) lost during boiling and the different impact of cooking approaches on vegetables structure, with the boiling procedure being more invasive in terms of structural pectin degradation in broccoli, and with a consequent higher washing-out effect of hydrophilic compounds (GLS) in boiling than steaming [49].

**Figure A1:**
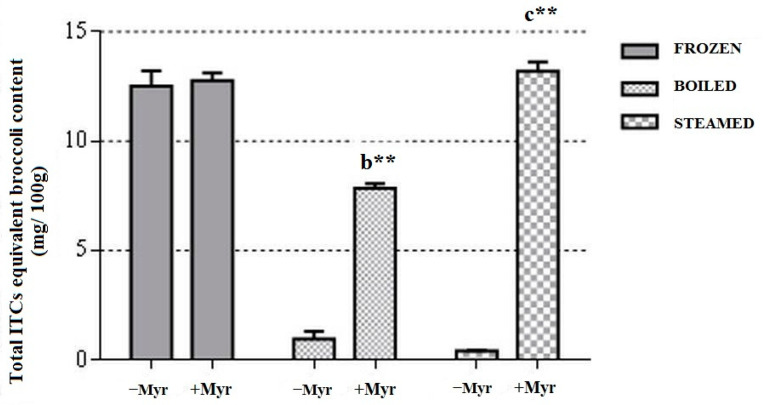
Total glucosinolates (GLS) levels converted to isothiocyanates with (+Myr) or without (−Myr) *Sinapis alba thioglucosidase* added to frozen (−20 °C for 15 days), boiled and steamed broccoli. Data are expressed as mg/100 g of vegetables ± SD of three technical replicates. ** *p* < 0.01 vs. boiled–Myr (b) and steamed–Myr (c).

In order to clarify a potential impact of residual enzymatic activity after thermal treatments, we also quantified total ITC produced without adding external *Sinapis alba thioglucosidase,* finding very low levels independently from cooking methods (Appendix A, Figure A1). The effect of heating treatments on denaturation of myrosinase of *Brassica* plants has been largely studied. In particular, Rungapamestry et al. [50] showed that myrosinase activity in cabbage is lost after microwaving for 2 min (−97%) or steaming for 7 min (−90%). 

Therefore, the plasma levels of total ITCs found during the 24 h following the intake of cooked broccoli originate exclusively from the hydrolytic activity of intestinal myrosinase. In fact, following consumption of broccoli with myrosinase inactivated, most of the ingested GLSs reach the colon, where they are hydrolyzed by the microflora to release isothiocyanates, along with other metabolites [51,52]. However, when myrosinase is active in the food ingested, GLSs are quickly hydrolyzed in the upper gastrointestinal tract, and the breakdown products are absorbed [53,54]. Accordingly, in our study the BroccoMax^®^ intake containing GLSs and active myrosinase resulted in a more rapid absorption of ITCs, peaking at 3 h compared to cooked broccoli (peaked at 6 h), even though the AUC calculated did not differ significantly from steamed vegetables. Our data highlight that the beneficial effects of dietary broccoli consumption are limited by the loss associated with cooking methods, in particular, boiling. Accordingly, the efficacy of broccoli as superfoods rich in glucosinolate is limited also in respect to the high daily consumption dose required to achieve biological significant concentration in the organism. On the other hand, supplementation with glucosinolate/myrosinase-enriched extracts (BroccoMax^®^, Jarrow Formulas, CA, USA) might provide a practical and efficient approach to the supplementation of these bioactive compounds. Comparing the intake of fresh broccoli and steamed, Conaway et al. [22] noticed that thermal treatment delayed the absorption of ITCs from the intestinal tract and, in contrast to our data, led to an increase in plasma availability three times lower than fresh vegetables, highlighting the key role of myrosinase in the conversion of GLSs to ITCs. Therefore, steaming treatment limited the loss of GLSs in broccoli cooking, thereby promoting a much higher bioavailability of isothiocyanates in plasma as compared to boiling. 

On the contrary, both cooking treatments affected positively the extractability of carotenoids. In particular, steaming led to a higher significant increase in lutein than β-carotene when compared to the raw vegetable. The outcomes confirmed that the effect of cooking on carotenoids depends on the level of tissue softening, which is considered higher in boiling water, provoking the release of matrix bound compounds. In accordance with these results, Gliszczyńska-Świgło et al. [32] found the boiling and steam-cooking of broccoli to provoke an increase in β-carotene and lutein with respect to raw broccoli. In the same way, Miglio et al. [55] found that the contents of all carotenoids significantly increased after the boiling (32%), as well as after the steaming (around 19%), of broccoli in comparison to the raw one. Many studies have been conducted on the effect of cooking on carotenoids in brassica vegetables (i.e., broccoli and cauliflower), also producing controversial findings and without considering if higher extractability of carotenoids is potentially translated to a higher plasma availability. In our study, the higher β-carotene and lutein extracted after both cooking techniques, with heat-transfer efficiency provoking different cell-disruption levels, did not affect their plasma bioavailability. However, our conclusion is limited to the fact that the carotenoid steady-state profile is the result of a long-term dietary pattern and is slightly influenced by acute supplementation with carotenoids-rich foods [37]. Moreover, our study is based on an acute intake of 4.8 mg of β-carotene and 4.8 mg of lutein for boiled broccoli, and of 2.3 mg of β-carotene and 2.6 mg of lutein for steamed broccoli. However, other authors investigated 4–11 days of similar levels of β-carotene and lutein from broccoli supplementation [2,37,38,39], registering in some cases plasma variations in carotenoids. Brown et al. [37] concluded that 600 g of broccoli had no significant effect on the plasma carotenoids concentration, in agreement with our outcomes. Granado et al. [2] reported a significant increase in plasma lutein concentrations after the intake for 1 week of 200 g broccoli, while some authors found a significant increase in both lutein and β-carotene in plasma after 4–10 days of broccoli supplementation [38,39]. 

Finally, phylloquinone, well-known for its beneficial role in blood coagulation and bone metabolism, represents the only molecule not affected by heat treatments. In accordance with our results, Lee et al. [35] noticed that boiling, blanching, microwaving and steaming did not alter significantly vitamin K1 content in broccoli. This is probably due to its chemical characteristics, as vit K1 is a fat soluble and relatively heat stable compound [43]. Similarly, plasma bioavailability did not differ from the type of cooking performed on the broccoli before supplementation. In fact, both the Area Under Curve and the maximum absorption peak, reached 6 h after ingestion, are very similar. These results are in agreement with some previous publications showing a comparable bioavailability curve of phylloquinone after the ingestion of kale [56,57,58], but they contrast with those of Garber et al. [59], who showed that phylloquinone peaked after 4 h by ingestion of spinach, reaching lower concentrations. A large number of variables undoubtedly affect the bioavailability of phylloquinone from the diet, i.e., specific foods, consumption of cooked vs. raw vegetables and fat content of the diet. A limitation of the study refers to the low sample size of the sample and the narrow age group of the subjects; however, the present experimental setting allows us to identify specific differences in the cooking methods with regard to bioactive compounds’ bioavailability

## 5. Conclusions

Steaming has demonstrated to be able to preserve GLSs in broccoli, thus promoting the plasma bioavailability of ITCs, as it resulted in being significantly higher than boiling and comparable to the plasma levels reached following the intake of a supplement containing glucoraphanin and active myrosinase. Therefore, considering the antioxidant action and the potential chemopreventive activity of ITCs, steaming represents the most suitable cooking method to promote the health benefits of broccoli in the diet. On the contrary, boiling favored a greater extraction of lipophilic antioxidants compounds, such as lutein and β-carotene, from broccoli, even if there was no increase in their levels after ingestion. Finally, both steaming and boiling did not change the phylloquinone content in the food, thus reflecting a similar increase in plasma levels for both cooking methods.

## Figures and Tables

**Table 1:** Amount (mg) of some bioactive compound given to volunteers by consuming 400 g of cooked broccoli (boiled and steamed) and total GLS administered by taking 3 capsules of BroccoMax^®^. Data are expressed as mean ± SD of five cooking replicates. Level of GLS in BroccoMax^®^ is as stated on the label by the manufacturer.

	**ITCs Equivalent**	**Phylloquinone**	**β-Carotene**	**Lutein**
BroccoMax^®^	90	-	-	-
Boiled	38.4 ± 4.4	1.36 ± 0.24	4.78 ± 0.03	4.83 ± 0.04
Steamed	56.8 ± 2.8	1.40 ± 0.72	2.26 ± 0.02	2.62 ± 0.03
	**GLS**			
BroccoMax^®^	90			

## Data Availability

The data is contained within the article.

## Appendix A

In order to verify if freezing and both cooking treatments were able to inhibit the endogenous myrosinase activity, a preliminary test was performed comparing the level of glucosinolates converted to ITCs before cyclocondensation reaction with or without Sinapis alba thioglucosidase incubation. Figure A1 emphasizes that keeping raw broccoli at −20 °C for 15 days did not affect endogenous myrosinase activity, as GLS levels converted to ITC are similar regardless of the addition of the enzyme. Conversely, the incubation with exogenous thioglucosidase is a key step to quantify glucosinolates originally present in cooked broccoli, because heating inactivated endogenous myrosinase prevents conversion to ITCs.
